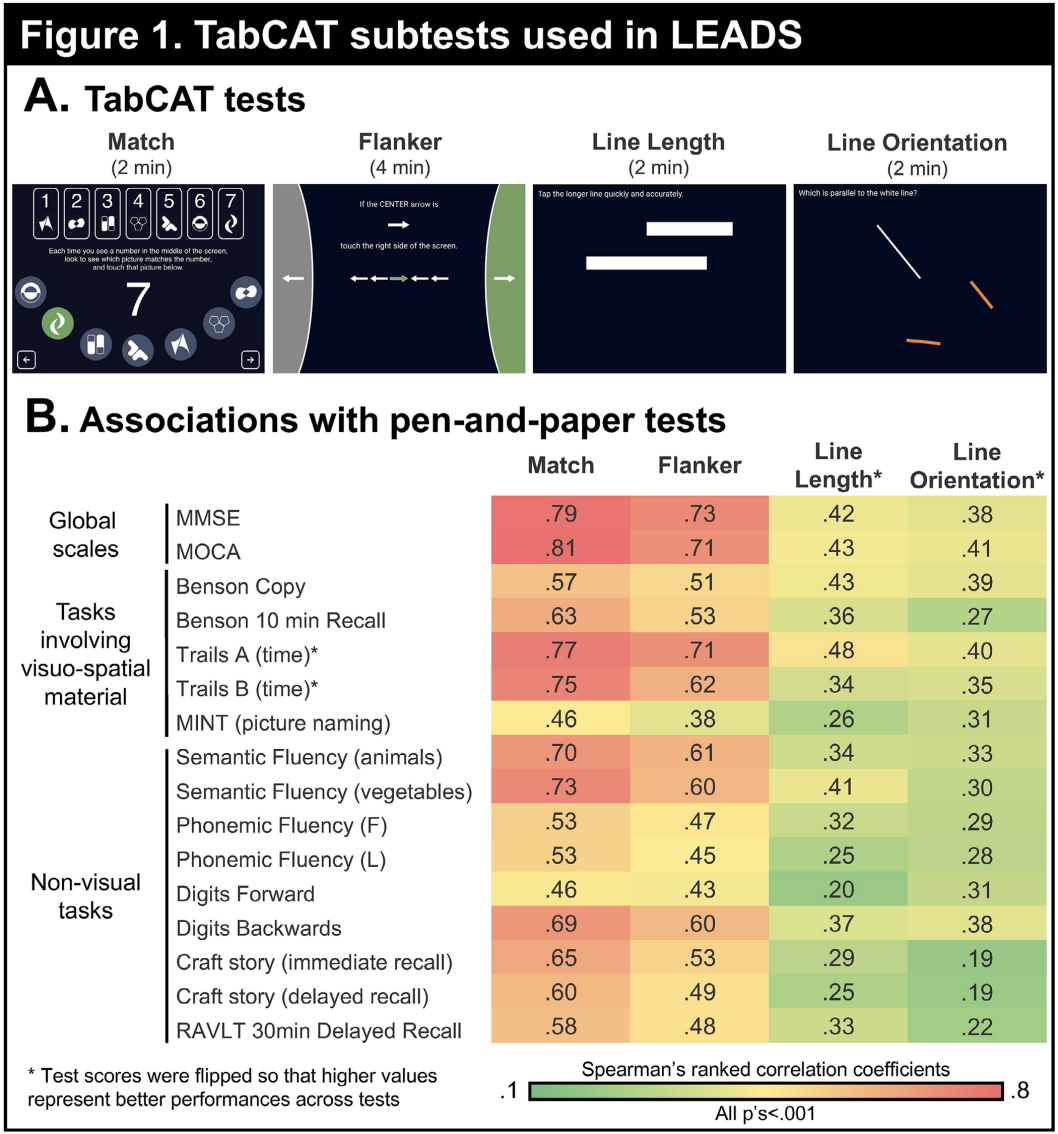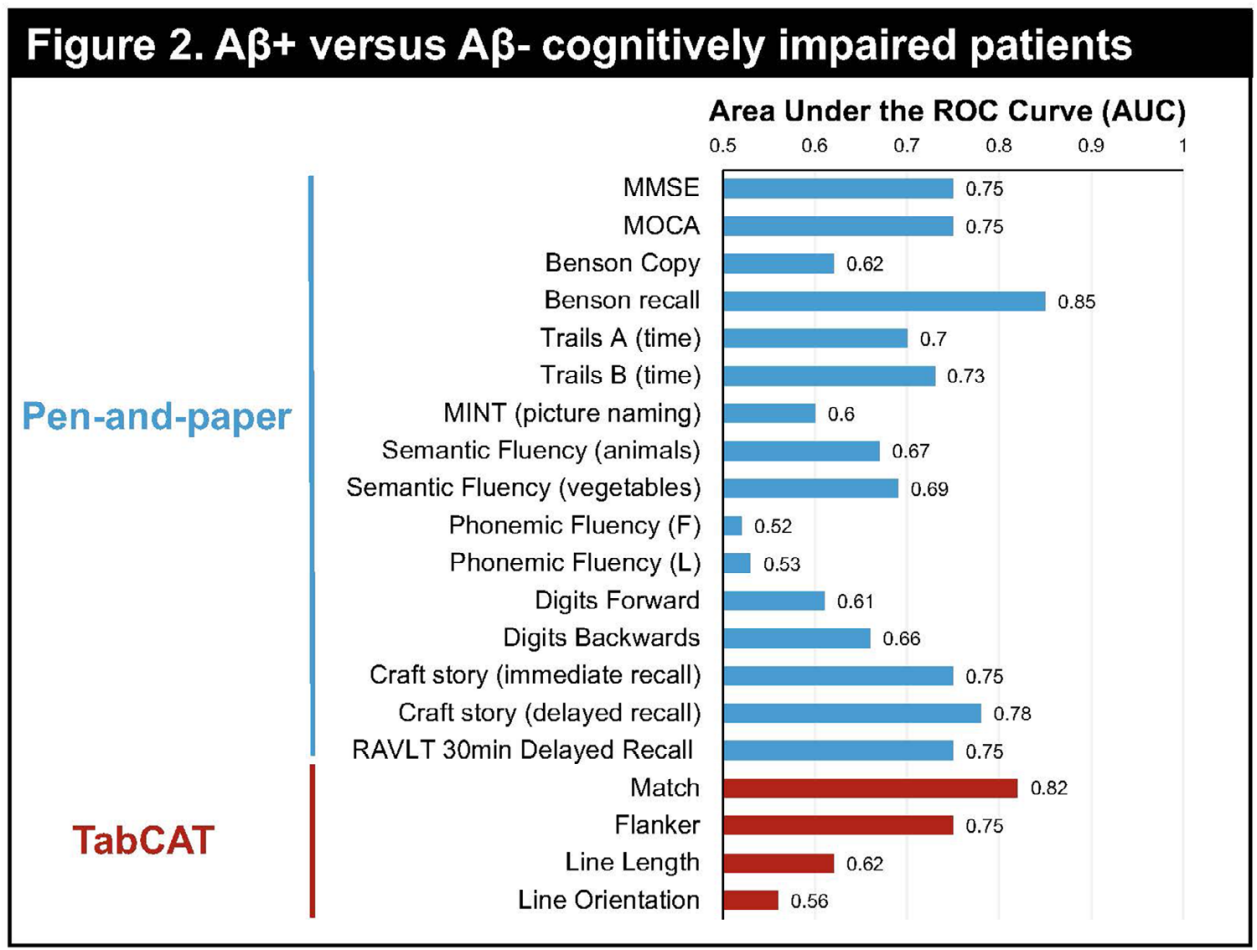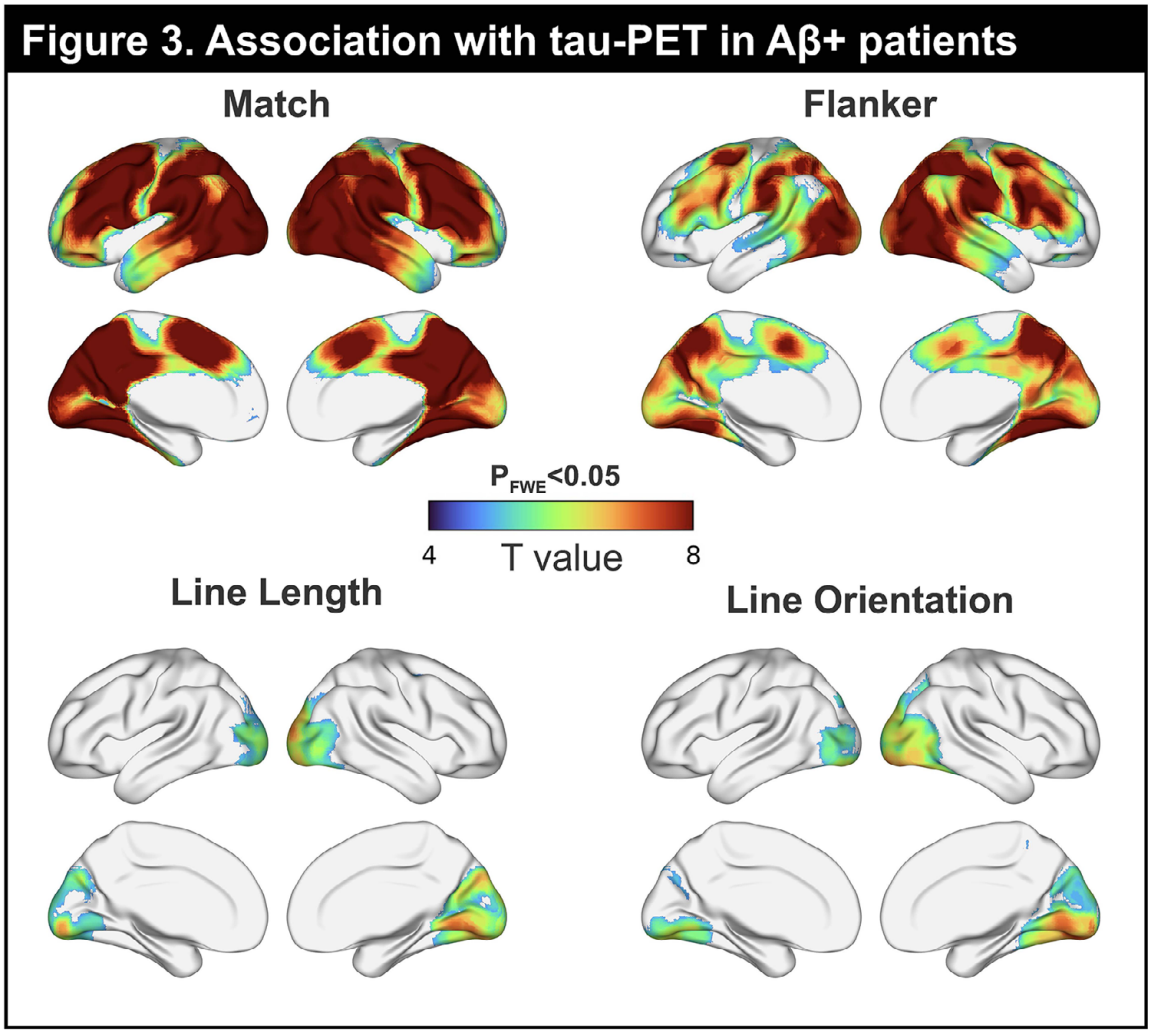# Tablet‐based cognitive assessment in a multisite study of early‐onset AD: association with pen‐and‐paper tests and PET measures of amyloid and tau

**DOI:** 10.1002/alz70857_104654

**Published:** 2025-12-25

**Authors:** W. Yembe Njamnshi, Konstantinos Chiotis, Elena Tsoy, Katherine L. Possin, Dustin B. Hammers, Ani Eloyan, Kala Kirby, Piyush Maiti, Ganna Blazhenets, Isabel Elaine Allen, Salma Rocha, Ranjani Shankar, Alinda Amuiri, Robert A. Koeppe, Maria C. Carrillo, Brad C. Dickerson, Liana G. Apostolova, Gil D. Rabinovici, Renaud La Joie

**Affiliations:** ^1^ Global Brain Health Institute, University of California, San Francisco, San Francisco, CA, USA; ^2^ Brain Research Africa Initiative, Yaounde, Center Region, Cameroon; ^3^ Memory and Aging Center, Weill Institute for Neurosciences, University of California San Francisco, San Francisco, CA, USA; ^4^ Memory and Aging Center, University of California San Francisco, San Francisco, CA, USA; ^5^ Memory and Aging Center, Weill Institute for Neurosciences, University of California, San Francisco, San Francisco, CA, USA; ^6^ Global Brain Health Institute (GBHI), University of California San Francisco (UCSF); & Trinity College Dublin, San Francisco, CA, USA; ^7^ Indiana University School of Medicine, Indianapolis, IN, USA; ^8^ Brown University, Providence, RI, USA; ^9^ Global Brain Health Institute, University of California, San Francisco, CA, USA, San Francisco, CA, USA, San Francisco, CA, USA; ^10^ Memory and Aging Center, UCSF Weill Institute forNeurosciences, University of California, San Francisco, San Francisco, CA, USA, San Francisco, CA, USA; ^11^ University of Michigan, Ann Arbor, MI, USA; ^12^ Alzheimer's Association, Chicago, IL, USA; ^13^ Massachusetts General Hospital/Harvard Medical School, Boston, MA, USA

## Abstract

**Background:**

People living with early‐onset Alzheimer's disease (EOAD) are often misdiagnosed or experience delays in diagnoses due to their young age of onset, non‐amnestic presentation, and rapid clinical progression. Brief digital cognitive measures may be a valuable and scalable alternative to traditional tests for early detection of EOAD. We examined the associations of the UCSF Tablet‐based Cognitive Assessment Tool (TabCAT) tests with clinical and pathophysiologic markers of AD in the multisite Longitudinal Early Onset Alzheimer's Disease Study.

**Method:**

83 cognitively unimpaired controls and 392 patients with a clinical diagnosis of EOAD (MCI or mild dementia) underwent TabCAT testing, including 2 tests of executive function/attention (Match, Flanker) and 2 visuospatial tests (Line Length and Line Orientation; Figure 1A). First, we assessed correlations between TabCAT scores and classic pen‐and‐paper tests in the whole cohort using ranked coefficients. Second, we used receiver operating characteristic analyses to examine each cognitive score's ability to distinguish between Aβ‐ (*n* = 95) and Aβ+ (*n* = 297) clinically impaired patients, as defined by a quantification‐aided visual read of Florbetaben‐PET. We then investigated regional associations between TabCAT scores and Flortaucipir (tau)‐PET using voxel‐wise analyses in the Aβ+ patients.

**Result:**

In the whole cohort, Match and Flanker were associated with all pen‐and‐paper tests, with strongest correlations observed with the MMSE, MoCA, and Trails A (*r*'s >0.7). As expected, Line Length and Line Orientation tests showed strongest correlations (*r*'s >0.4) with traditional tasks that rely on visuospatial skills: Benson Figure Copy and Trails A (Figure 1B). Of TabCAT tests, Match best discriminated Aβ+ from Aβ‐ patients (Area Under the Curve (AUC)=0.82), outperforming the MMSE (AUC=0.75) and MoCA (AUC=0.75), and comparable to the overall best performing test Benson Figure Recall (AUC=0.85; Figure 2). In Aβ+ patients, Match and Flanker correlated with tau‐PET signal in most cortical areas, especially dorsal frontal and parietal areas. The Line tests were specifically associated with occipital tau‐PET signal (Figure 3).

**Conclusion:**

In this large multisite study, TabCAT measures show good concurrent validity and associations with pathophysiological markers of AD: Aβ‐PET positivity and neuroanatomical specific patterns of tau‐PET. These digital measures have promise for frontline identification and early diagnosis of patients with EOAD.